# Relation between parenting style and confident decision-making in a student population

**DOI:** 10.1371/journal.pone.0302495

**Published:** 2024-11-12

**Authors:** Kara Wolf, Zuzanna Tajchman, Iris Vilares

**Affiliations:** Department of Psychology, University of Minnesota, Minneapolis, MN, United States of America; National University of Medical Sciences, PAKISTAN

## Abstract

Parenting styles differ in their expression of care and (over)protection behaviors and are associated with markedly different outcomes in children. While research has examined the effects of parenting styles on global self-esteem and self-efficacy, little is known about how they might relate to decision-confidence (metacognitive bias) in a task. This study examined the possible relation between students’ decision-confidence and the perceived parenting style of their primary caregiver. Participants (*N* = 246) played a trust game and rated their confidence in task choices. The perceived parenting style of their caregiver was determined using participants’ responses to the Parental Bonding Instrument. Participants’ decision-confidence was not significantly associated with their caregiver’s parenting style. However, decision-confidence was positively related with self-efficacy to a small degree. Exploratory analyses indicated that participants with overprotective caregivers were more likely to report poor self-efficacy and self-esteem; increased depression, anxiety, and stress symptoms; childhood adversity; and anxious and avoidant attachment; while participants with caring caregivers showed the opposite pattern. Our results are a step towards understanding the potential effects of parenting on adult children’s decision-confidence and contribute to growing evidence that perceived parental behaviors have strong associations with young adults’ mental health, self-worth, and relationship attachment.

## Introduction

The nature-versus-nurture debate highlights the influence of parenting behaviors on a child’s psychosocial development and later dispositions. Historically, social theorists have separated parenting behaviors into two bipolar dimensions. Although the names of the dimensions vary depending on the theorist, they foundationally refer to the same two constructs of “care” and “(over)protection”.

The care dimension is bounded at the positive end by parental acceptance, empathy, and responsiveness, and on the opposing end by emotional coldness, rejection, and indifference. At one end of the overprotection dimension is parental encouragement of autonomy, and at the other is strictness and control [1].

Prior to the conception of these dimensions, Baumrind [2] developed a typology for parenting styles using home observations and interviews of parents and preschool-aged children, creating authoritative, authoritarian, and permissive styles. Maccoby and Martin [3] later devised behavioral dimensions called “responsiveness/support” and “demandingness”, constructs that are interchangeable with the care and overprotection dimensions previously mentioned [4]. Authoritative parents were classified as highly caring and protective, while authoritarian parents were uncaring but overprotective, and permissive parents were highly caring but under-protective. A fourth style, uninvolved parenting, was added by Maccoby and Martin [3] to include a parent who is uncaring and under-protective. Our study uses their terminology to make comparisons with contemporary research on parenting styles; however, our primary measure, the Parental Bonding Instrument [5], utilizes self-report data from adult children and different nomenclature.

The parenting styles from the Parental Bonding Instrument (PBI) differ from Maccoby and Martin’s four styles [3], but have similar behavioral dimensions [4]. Thus, the affectionate constraint style from the PBI is akin to the authoritative style, the affectionless control style is akin to the authoritarian, the optimal style is akin to the permissive, and the neglectful style is akin to the uninvolved style [4].

Each of the aforementioned styles has discrete implications for children’s psychosocial development that may extend into adulthood [6, 7]. To illustrate, children of authoritative parents exhibit positive outcomes that are reflected in their overall health, educational achievements, emotional well-being, and behavioral adjustment [1, 8]. In contrast, children reared by authoritarian and uninvolved parents display negative self-perceptions, with children of uninvolved parents also exhibiting behavioral maladjustment [6, 9, 10]. The children of permissive parents experience more adaptive emotional outcomes than children of authoritarian and uninvolved parents, but not as substantial as those of children of authoritative parents [8, 9]. However, a study by Garcia and Gracia [7] demonstrated that permissive parenting was associated with the best outcomes in a large sample of teens from Spain, indicating that the ideal parenting style might vary between cultures.

### Confidence in decision-making

The lack of research examining parenting behavior and decision-making confidence is surprising, given that psychosocial trait development is thought to begin with the child-rearing environment [11]. Decision-confidence can be defined as an individual’s self-assurance in their judgment following a decision [12]. A decreased sense of decision-confidence can reflect a tendency towards indecisiveness or a delay in making a decision. Also called metacognitive bias, decision-confidence is subjective, and can be independent of actual performance [13].

Parents regulate their children’s decisions to varying degrees [8, 14]. Authoritative parents are democratic, allowing their children to make guided decisions [14, 15]. They are present in their child’s environment but not overbearing, and allow their child to learn from their mistakes, enabling them to grow into high-achieving and behaviorally well-adjusted adults [8, 15]. Authoritarian parents, exercising highly overprotective behaviors, frequently dictate decisions without including their youngsters [14, 15]. They expect compliance and create controlled environments with strict rules [8]. Their children are typically obedient but suffer from low self-esteem and social competence. Conversely, permissive parents are under-protective and allow decisional freedom [10]. They tend to show concern but avoid boundaries and confrontation, often resulting in their children displaying problems with behavioral and emotional regulation [8, 15]. Uninvolved parents also allow more decisional freedom due to a lack of engaging in parental responsibilities [8]. They are rejecting and neglectful; subsequently, their offspring often develop behavioral and self-esteem issues and are less achieving than their peers [8, 15]. Thus, we expected parenting style to potentially affect a child’s decision-confidence.

The objective of this study was to investigate whether an individual’s decision-confidence could be related to the parenting style of their primary caregiver, based on the knowledge that environments created by parents vary in their democratic structure and emotional support [9, 14].

We hypothesized that adults who perceived their parent as authoritative would report more confidence in their decisions during a structured task than participants reared by any other style, due to the democratic guidance they received with decisions from a supportive and present parent. We anticipated that individuals with perceived permissive parents would also exhibit high decision-confidence, but secondary to those with authoritative parents, due to permissive parents’ care but lack of involvement. Individuals who reported authoritarian or uninvolved parents were expected to rate the lowest confidence in their decisions, with no significant difference between the two styles.

Confidence in decision-making has been linked to an individual’s self-esteem, self-efficacy, depression, anxiety, and attachment style in various contexts [16–20]. These psychosocial variables have also been linked to parenting styles [11, 21–23]. Consequently, our study considers each of these variables to differentiate their connection to decision-confidence from any association with parenting style. Rather than exist as confounds to the potential effect of parenting style on decision-making, they may drive an association between the two.

Thus, as secondary hypotheses, intended to clarify previous research, we predicted that individuals with low self-reported self-esteem and self-efficacy would make less confident decisions than those with high self-esteem and self-efficacy. We also hypothesized that participants who reported adverse childhood experiences (ACEs) and symptoms of depression, anxiety, and stress would be less confident in their decisions than those who had not, and that individuals with anxious or avoidant attachment tendencies would express less decision-confidence than those with secure attachment.

## Method

### Participants

Participant demographics are described in Table A in the S1 File. The sample consisted of 246 undergraduate psychology students recruited from the University of Minnesota. Non-random voluntary response sampling was used for this project due to the global COVID-19 pandemic, which had shut down all non-essential operations at the university. Furthermore, as the relation between parenting style received and future decision-confidence has been largely unexplored, we opted to first use a convenience sample, provided there would be enough representation of at least two parenting styles for analyses to be performed (see sample size rationale below).

259 participants completed the study, but the data of 13 were excluded from the analysis because they failed the attention checks (6), did not correctly identify a primary caregiver (5), or were put into the care of a non-parent caregiver after age one (2). We preregistered that we would exclude the data of participants who passed less than 80% of our attention checks, assuming they answered the study at random. Of the six participants that failed attention checks, three were reared by an optimal/permissive caregiver, two by an affectionless control/authoritarian caregiver, and one by an affective constraint/authoritative caregiver. The parenting style of the remaining seven excluded participants could not be determined, thus providing reason for their exclusion, as the Parental Bonding Instrument asks participants to reflect on a single caregiver’s behaviors from the participant’s birth to age 16. Five participants did not identify a caregiver per questionnaire instructions, and two participants were not reared by at least one consistent caregiver from birth age to 16.

### Procedure

This study was approved by the University of Minnesota’s Institutional Review Board (STUDY00005811) and preregistered prior to data collection (https://osf.io/xp2ed/?a21d65fd6292475ba1ee8b2053f76117). Written informed consent was obtained from all subjects before study onset.

The Parental Bonding Instrument [PBI; 5] was used to determine the parenting style of participants’ primary caregiver—identified by participants as the person who most engaged in parenting behaviors with them from birth to age sixteen. Participants also responded to the New General Self-Efficacy scale [NGSE; 24], Rosenberg Self-Esteem Scale [RSES; 25], Adverse Childhood Experiences scale [ACE; 26], Depression, Anxiety, and Stress Scale-21 [DASS-21; 27], and the Adult Attachment Questionnaire [AAQ; 28]. All measures are publicly accessible.

Participants then completed the Trust Game [29], the behavioral task during which they rated their confidence in task decisions (see Supplementary Methods in S1 Supporting Information for game instructions). The Trust Game is a widely used behavioral task within cognitive psychology, neuroscience, and economics used to measure trust and reciprocity [30–32]. However, for the purpose of this study, it served as a vehicle for measuring decision-confidence. Participants made socio-economic decisions, such as whether to invest a certain amount of monetary units to a stranger—not knowing if they would receive any in return—and then rated their confidence in their decisions. While decision-confidence (metacognitive bias) is more commonly studied using perceptual or knowledge-based tasks, researchers have recently started to assess decision-confidence in the Trust Game [33, 34]. Furthermore, Torunsky and Vilares [33] found that decision-confidence measured during the Trust Game was strongly correlated with decision-confidence measured during a perceptual and an emotional task. Of these tasks, the Trust Game is the only that involves anticipating the behavior of a “partner” that creates social uncertainty, and thus is more relevant to our study’s question of whether decision-confidence could be linked to the parenting environment (a social setting).

#### Sample size rationale

A power analysis using G*Power software was conducted to determine our ideal sample size. A one-way ANOVA using a medium effect size (0.25) with alpha = .05 and .80 power required at least 180 participants to test our principal hypothesis, with 45 participants from each of the four parenting-style groups (or 53 per group from three parenting-style groups to result in 159 participants total, or 64 per group from two parenting-style groups to result in 128 participants total). Consequently, we preregistered that we would collect data from 210 participants to accommodate potential exclusions.

Our final sample size was 246, as we extended our collection timeline to obtain enough participants per group to conduct an ANOVA. Subsequently, the recruitment period for this study ran from March 30^th^-April 30^th^, 2021 and reopened from June 26^th^-August 15^th^, 2021.

### Analysis

We planned to conduct a one-way ANOVA with perceived parenting style as the predictor variable and mean decision-confidence rating as the dependent variable to test whether decision-confidence significantly differed between parenting styles. All four perceived parenting styles were represented in the data (authoritarian/affectionless control, *n* = 62; permissive/optimal, *n* = 107; authoritative/affectionate constraint, *n* = 56; uninvolved/neglectful, *n* = 21). However, the uninvolved parenting group was not large enough to match any criterion from the power analysis calculations for this study, so only the authoritarian/affectionless control, permissive/optimal, and authoritative/affectionate constraint styles were compared.

Our sample slightly violated the normality assumption for a one-way ANOVA to examine potential differences in mean decision-confidence among parenting style groups, so the nonparametric Kruskal-Wallis one-way ANOVA on ranks test was performed to determine if any significant difference existed between the mean ranks of decision-confidence scores of students reared by the three separate parenting styles that had enough data (authoritarian/affectionless control, permissive/optimal, and authoritative/affectionate).

To examine how decision-confidence related to our additional variables (PBI perceived parental care and overprotection; NGSE self-efficacy; RSES self-esteem; ACE adverse childhood experiences; DASS-21 [depression, anxiety, and stress] symptoms; and AAQ attachment avoidance and anxiety), we performed Spearman correlations using mean decision-confidence ratings and scores obtained from the six self-report measures. Our sample met the assumptions for Spearman correlations prior to completing these tests.

To understand the relative importance of these predictors, we planned to conduct a multiple linear regression with scores from the above six self-report measures as predictor variables and mean confidence ratings as the dependent variable. However, after analyzing the assumptions for the multiple linear regression, only the sampling distribution of the residuals of scores for attachment avoidance (*p* = .07) and attachment anxiety (*p* = .64) passed the Shapiro-Wilk normality test, so the multiple regression was not completed.

#### Exploratory analysis

In the preregistration, we hypothesized how decision-confidence would relate to the DASS-21 overall score, but not for each of the subscores. Thus, we conducted three additional exploratory Spearman correlations comparing mean confidence ratings and the DASS-21 depression, anxiety, and stress symptoms subscores.

See Supplementary Methods in S1 Supporting Information for our remaining exploratory analysis plan.

## Results

The dataset generated during this study is publicly available [35].

### Descriptive statistics

Mean scores on all dependent measures are included in Table B in S1 File.

### Parenting style and decision-confidence

**Fig 1:**
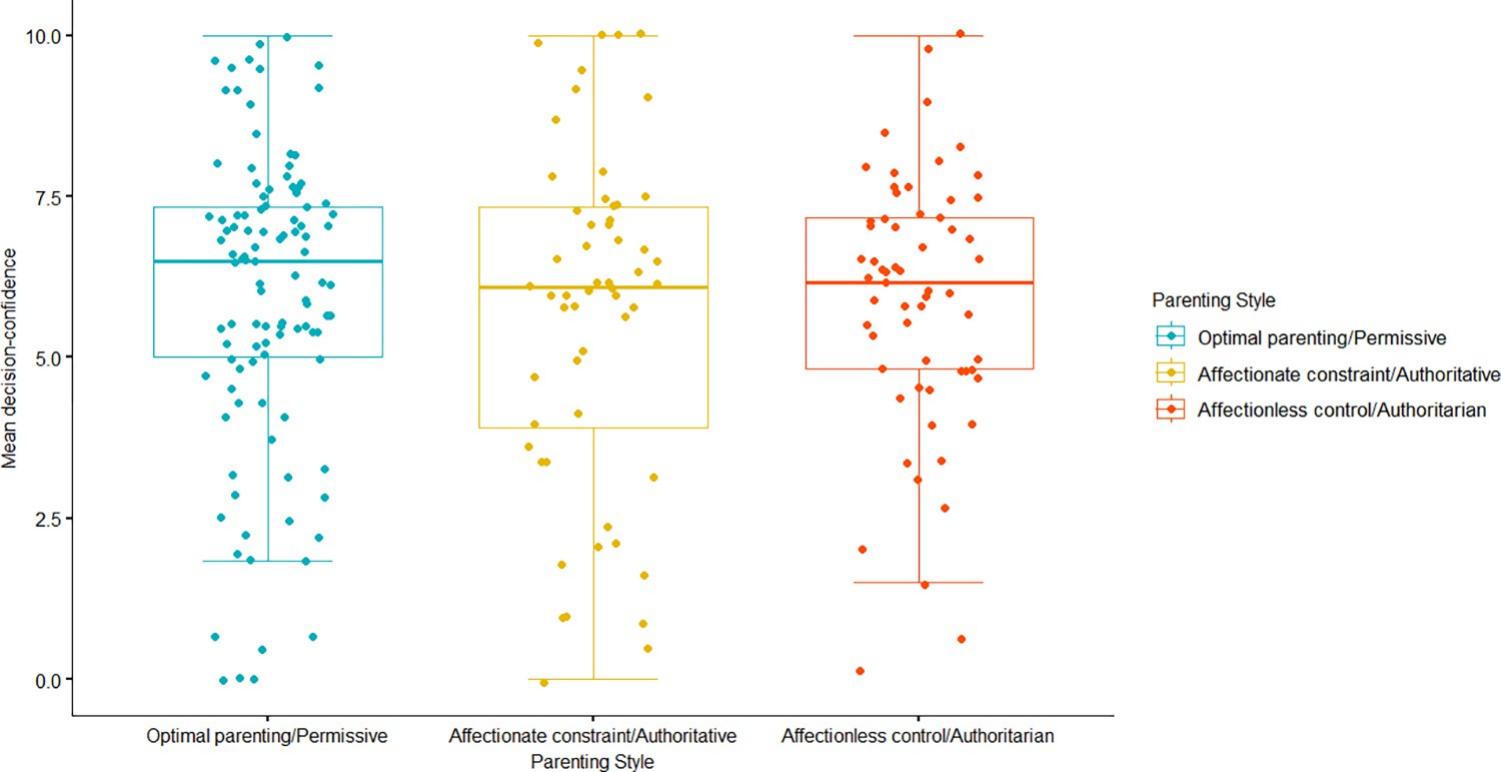
Boxplot depicting the non-significant difference between mean ranks of decision-confidence scores for parenting style groups. *Note*. Decision-confidence was determined by mean confidence ratings made by participants during the Trust Game task, and perceived parenting style was determined by care and overprotection scores obtained from the Parental Bonding Instrument. Error bars represent a 95% confidence interval.

Our sample slightly violated the normality assumption for a one-way ANOVA to examine potential differences in mean decision-confidence among parenting style groups, so the nonparametric Kruskal-Wallis test was performed to determine if any significant difference existed between the mean ranks of decision-confidence scores of students reared by three separate parenting styles (authoritarian/affectionless control, permissive/optimal, and authoritative/affectionate). No significant difference was found in the mean ranks between parenting style groups for decision-confidence (*H*(2) = 0.31, *p* = .86; see Fig 1).

Our sample met the assumptions for Spearman correlations prior to completing these tests. Decision-confidence was not significantly correlated with the behavioral dimensions from the Parental Bonding Instrument—perceived parental care (*r*_*s*_ = .01, *p* = .84, *N* = 246, Spearman correlations) nor overprotection (*r*_*s*_ = -.03, *p* = .64, *N* = 246; see Table 1). Thus, no relation was found between the perceived parenting style an individual experienced in childhood and their decision-confidence throughout the Trust Game task.

**Table 1:** Spearman correlations with decision-confidence and study variables.

Variables	*N*	Decision-confidence	*p*
1. Parental care	246	.01	.844
2. Parental overprotection	246	-.03	.636
3. Self-efficacy	246	.13	.049
4. Self-esteem	246	.08	.210
5. Adverse childhood experiences	238^a^	-.03	.594
6. DASS-21 overall	246	-.11	.082
7. Depression symptoms^b^	246	-.13	.044
8. Anxiety symptoms^b^	246	-.08	.239
9. Stress symptoms^b^	246	-.10	.123
10. Attachment avoidance	246	-.05	.425
11. Attachment anxiety	246	-.08	.193

*Note*. Variables were measured by the Parental Bonding Instrument; New General Self-Efficacy scale; Rosenberg Self-Esteem Scale; Adverse Childhood Experiences scale; Depression, Anxiety, and Stress Scale-21 (“DASS-21 overall” represents combined depression, anxiety, and stress symptoms while symptom subscales are also listed); and the Adult Attachment Questionnaire. Decision-confidence was measured using confidence ratings from a behavioral task.

^a^Participants could skip the Adverse Childhood Experiences scale due to its sensitive questions; consequently, *N* = 238 for this test.

^b^Exploratory analysis, see Table 2 for full matrix of exploratory correlations.

### Decision-confidence and secondary variables

Decision-confidence was also not significantly related to the majority of the secondary outcomes we measured: self-esteem, adverse childhood experiences, overall DASS-21 symptom scores, attachment avoidance, nor attachment anxiety (see Table 1). However, a small but significant positive correlation was found between decision-confidence and self-efficacy (*r*_*s*_ = .13, *p* = .049, *N* = 246).

### Exploratory analysis

#### Correlation between decision-confidence and depression, anxiety, and stress symptoms

We pre-registered our hypothesis that decision-confidence would be negatively related to DASS-21 symptoms, but did not specify that we would include symptom subscores in our analysis. Additional Spearman correlations with the subscores revealed that decision-confidence was not significantly correlated with anxiety (*r*_*s*_ = -.08, *p* = .24) or stress symptoms (*r*_*s*_ = -.10, *p* = .12), but decision-confidence was slightly negatively correlated with depressive symptoms (*r*_*s*_ = -.13, *p* = .044, *N* = 246; see Table 1). Although this correlation was in the expected direction, it would not reach significance if completing a Bonferroni correction for multiple comparisons; thus, it should be taken as merely suggestive.

#### Parenting style groups and secondary variables

Exploratory Kruskal-Wallis tests displayed significant differences between mean ranks of scores of participants, based on the parenting style they were reared by, for self-efficacy (*H*(2) = 29.14, *p* < .001); self-esteem (*H*(2) = 38.64, *p* < .001); adverse childhood experiences (*H*(2) = 39.01, *p* < .001; see Table C in S1 File); depression, anxiety, and stress symptoms (*H*(2) = 38.77, *p* < .001); attachment avoidance (*H*(2) = 36.76, *p* < .001); and attachment anxiety (*H*(2) = 23.13, *p* < .001). See Supplementary Results and Table D in S1 File for the post hoc analysis of parenting style group differences.

#### Correlations between parenting behaviors and secondary variables

Perceived parental care and overprotection scores were significantly correlated with all variables aside from decision-confidence (see Table 2). Perceived parental care was positively correlated with self-efficacy and self-esteem (*p* < .001), and negatively correlated with adverse childhood experiences, scores on the overall DASS-21, attachment avoidance and anxiety, and perceived parental overprotection scores (all *p* < .001). Correlations with perceived parental overprotection showed the exact opposite pattern (see Table 2).

**Table 2:** Spearman correlation matrix featuring study variables and exploratory results.

Variables	1	2	3	4	5^a^	6	7	8	9	10	11
1. Parental care	--										
2. Parental overprotection	-.54***	--									
3. Self-efficacy	.44***	-.31***	--								
4. Self-esteem	.49***	-.41***	.69***	--							
5. Childhood adversity^a^	-.46***	.32***	-.21*	-.37***	--						
6. DASS-21 overall	-.44***	.45***	-.43***	-.68***	.46***	--					
7. Depression symptoms	-.42***	.39***	-.44***	-.71***	.45***	.89***	--				
8. Anxiety symptoms	-.39***	.43***	-.38***	-.58***	.38***	.90***	.71***	--			
9. Stress symptoms	-.41***	.42***	-.35***	-.58***	.43***	.93***	.74***	.79***	--		
10. Attachment avoidance	-.49***	.39***	-.38***	-.49***	.46***	.51***	.53***	.46***	.43***	--	
11. Attachment anxiety	-.33***	.31***	-.36***	-.50***	.30***	.54***	.53***	.47***	.49***	.38***	--

*Note*. Variables were measured by the Parental Bonding Instrument (perceived parental care and overprotection subscales); New General Self-Efficacy scale; Rosenberg Self-Esteem Scale; the Adverse Childhood Experiences scale; Depression, Anxiety, and Stress Scale-21 (“DASS-21 overall” represents the combined symptoms of depression, anxiety, and stress while symptom subscales are also listed); and the Adult Attachment Questionnaire (attachment avoidance and anxiety subscales).

^a^Participants were able to skip the Adverse Childhood Experiences scale due to the sensitive nature of its questions. The data of eight participants were removed via pairwise deletion from any correlational test involving the ACE scale (such that *N* = 238), but are represented in the other tests (*N* = 246).

*p < .05.

***p < .0001. Note that all correlations marked with a *** would remain significant even after doing a Bonferroni correction for multiple comparisons.

Furthermore, self-efficacy; self-esteem; quantity of adverse childhood experiences; depressive, anxiety, and stress symptom scores (overall and individually); and attachment avoidance and anxiety were all significantly correlated with one another (see Table 2).

## Discussion

### Hypotheses

The purpose of this study was to gain a better understanding of the link or possible variations between perceived parenting style and decision-confidence in adults. Our results found no correlation between an individual’s decision-confidence in a trust game task and the parenting style of their primary caregiver.

Further investigation revealed no link between the two behavioral dimensions of parenting styles—perceived parental care and perceived parental overprotection—and decision-confidence. Neither was decision-confidence associated with self-esteem; self-efficacy; symptoms of anxiety or stress; adverse childhood experiences; nor attachment. There was a small correlation between decision-confidence and self-efficacy in the expected direction (positive), and exploratory analyses revealed a small negative correlation between decision-confidence and depression symptoms. Nevertheless, both of these associations were of small magnitude and would not survive correction for multiple comparisons, so they should be viewed as merely suggestive.

However, exploratory analyses confirmed that self-esteem, self-efficacy, adverse childhood experiences, symptoms of psychological distress, and attachment were all associated with perceived parental care and overprotection, and that each of these variables was interrelated with one another. Furthermore, groups differed based on the parenting style of their primary caregiver for each of these developmental outcomes in a manner that reflects previous research (see Supplementary Discussion in S1 Supporting Information).

### Decision-confidence and the self-concept

Decision-confidence was defined as a feeling of self-assurance following a decision [6]. In studies by Campbell [13] and Vancouver et al. [19], an individual’s self-esteem and self-efficacy was related to their confidence ratings following decisions made during an assessment. Curiously, our study did not find an association between participants’ decision-confidence and self-esteem, but a potential link between their decision-confidence and self-efficacy only. Considering that self-esteem reflects a person’s global judgment of their worth [13] while self-efficacy measures one’s sense of competency in their abilities [16], it is possible that participants’ reported self-efficacy related more to decision-confidence in our study. However, the relation between self-efficacy and decision-confidence found in this study was not strong.

### Decision-confidence and mental health

We were surprised that participants who lacked confidence in their Trust Game decisions did not have poorer mental health, except perhaps for depression-like symptoms. Participants with depressive symptoms appeared to lack decision-confidence, but those with anxious symptoms did not, the latter contrasting with previous research [14, 15]. This is curious, considering low decision-confidence is implicated in a common symptom of depression and anxiety—indecisiveness [36]—and that depression and anxiety symptoms were strongly linked in our study. Indecisive individuals tend to lack confidence in making decisions [36]. Perhaps participants with depressive symptoms experienced more indecision in this study, given that they also reported slightly lower self-esteem and self-efficacy than those with anxiety symptoms. Nevertheless, the connection between depressive symptoms and low decision-confidence was not strong, and future studies are needed to corroborate these findings.

It was furthermore surprising that participants who reported adverse childhood experiences were not less confident in their decisions than those who did not. There is evidence that survivors of childhood abuse experience poor mental health outcomes [37], including low self-esteem [38], which is related to one’s confidence in their cognitive abilities [19]. In our study, participants who endured childhood adversity in our study did report lower self-efficacy and self-esteem than those who did not. It is possible that decision-confidence in our task was not representative of one’s overall confidence; however, there is evidence of decision-confidence in the Trust Game correlating with confidence in other tasks, implicating its potential to be a stable construct [33]. Perhaps the lack of association between childhood adversity and decision-confidence is influenced by social support from one nurturing parent or another adult, as Cheong and colleagues [39] suggest that perceived social support may prevent a survivor of childhood trauma from developing mental health issues. Attachment theory also asserts the importance of key early relationships to later social-emotional functioning [22]; nonetheless, in this study, attachment security from social support was not significantly associated with an individual’s task decision-confidence.

### Parenting and overall outcomes

In this study, exploratory analyses found that permissive/optimal parenting was associated with the most confident and well-adapted kids (in terms of higher reported self-esteem; lower depression, anxiety, and stress symptoms; and attachment avoidance), but other research has implicated authoritative parents to be superior for these effects [10, 40]. We confirmed that participants who identified authoritarian, uncaring, and overprotective parents were more likely to report low feelings of self-worth and high psychological distress, which appears to be true even in studies that solely measured parental control [23, 41]. However, our sample consisted of predominantly white, female students from a Western university; therefore, it is possible that an authoritarian parent may be experienced less negatively by individuals of another culture or age group. Consequently, these exploratory results cannot be generalized, and further research is necessary to confirm these findings.

Additionally, our study cannot support that parenting has an impact on decision-confidence in the Trust Game, as parental care alone, while linked to many positive psychosocial outcomes, was not associated at all with decision-confidence in our task. Indeed, high care combined with close supervision is crucial for adolescents to make safer and smarter choices [42], but we were interested in whether parenting behaviors were associated with how confidently young adults make decisions, independent of whether they could be considered objectively “good” ones.

### Limitations

There are several limitations to this study that merit consideration. The first was a lack of representation in our sample—considering participants were recruited from a Western university’s psychology program, the sample was predominantly white and female; therefore, our results cannot be generalized. While research suggests that authoritarian parenting (low care; high overprotection) is generally associated with negative outcomes in white samples, this may not be true for other races/ethnicities [10, 43]. Future studies with larger, diverse samples should further examine the association between parental variables and ethnic background.

Furthermore, our sample was not only ethnoculturally homogeneous, but it was also socially homogeneous. All of our participants were college-educated students, and 82% were raised in a two-parent household. Regrettably, we had to exclude two participants who had been put into the care of an adoptive caregiver after the age of one because of the limitations of our methodology for determining parenting style—the Parental Bonding Instrument required participants to report on a sole caregiver from birth to age 16. The lack of representation of individuals reared in a single-parent household or individuals reared by an adoptive caregiver in our sample could result in a lack of representation of individuals who have grown up stressed, disadvantaged, or with adverse childhood experiences, thus affecting our internal validity.

Secondly, our method of measuring confidence could potentially be challenged. The Trust Game may not have been the ideal task for engagement, considering that participants responded to hypothetical, online scenarios due to the global COVID-19 pandemic instead of playing live with a partner. As such, participants’ potential indifference to their choices could have affected decision-confidence validity. Confidence ratings from the Trust Game may have also reflected participants’ lack of comprehension of game rules, despite a pre-task quiz to check their understanding. A study by Torunsky and Vilares [33] demonstrated that decision-confidence in the Trust Game correlates with confidence measured in other tasks, suggesting a stable factor for decision-confidence; however, future research might use a different behavioral task or set of tasks to examine decision-confidence.

Moreover, it is possible that the strength of the association between parenting and decision-confidence is small, and there was not enough variance in our population for a significant result. A majority of participants were reared by one of two parenting styles associated with the most positive outcomes, the permissive/optimal and authoritative/affectionate constraint styles, possibly a result of our sampling bias mentioned above. The parenting styles associated with more negative outcomes in children, the authoritarian/affectionless control and uninvolved/neglectful styles [23], combined reared less than a third of participants. Thus, a more diverse sampling of the extreme ends of parenting behaviors may better highlight an association between parenting styles and decision-confidence if there is one to be found.

Another limitation of this study was the single method used to determine perceived parental style. The Parental Bonding Instrument utilized self-report data from individuals reflecting on one parent’s behavior. While the PBI is accepted as a reliable and valid measure of perceived parenting with long-term retest stability [44], future studies could corroborate this measure with additional assessments, such as parent interviews or sibling and parent reports.

Additionally, participants in this study were asked to identify a single primary caregiver, and 88% chose their mother. It is worth noting there may be differences in outcomes related to perceived paternal versus maternal behavior [40]. The PBI acknowledges this by providing different cut-off scores for care and overprotection based on whether the instrument was completed referring to a maternal or paternal figure. However, some parents fall close to these scores and may resemble an “average” level of care or overprotection rather than a “high” or “low” designation. To account for this, we examined how care and overprotection correlated with the remaining variables in case a relation existed that was not clear when investigating differences between parenting styles. Finally, different ages may be associated with different “optimal” parenting strategies. Younger children may benefit from more protection, while overprotection may be experienced negatively by teens. Future studies could use several different forms of assessment as well as questionnaires related to parental behavior to see which associations exist.

Furthermore, our study is a cross-sectional study; therefore, the direction of causality cannot be established if there is one to be found. For example, it is quite possible that the higher number of adverse childhood experiences (ACEs) reported by some of our participants drives both the effects on parenting as well as the negative effects on mental health. Indeed, research indicates that there is an intergenerational component of ACEs—children of parents that suffered higher number of ACEs are more likely to experience ACEs themselves and, furthermore, experiencing ACEs can affect both mental health and subsequent parenting behavior [43, 45–48]. Other factors, such as socio-economic status, may also play a role. While random allocation to different parenting styles or ACEs cannot and should not be done, well-designed longitudinal studies can potentially examine the causal relationships among these variables.

Finally, our pre-registered hypothesis was only concerned with the relation between parenting and decision-confidence. Thus, the relations found between parenting and the other psychosocial variables in this experiment, while generally in agreement with the existing literature, should be taken as exploratory, and future studies are needed to confirm these findings.

## Conclusions

Despite these limitations, our research can be viewed as a step toward examining a potential effect of parenting style on adult’s decision-confidence, as obtained from a behavioral task and not solely a questionnaire measure. Our exploratory findings also contribute to growing evidence that perceived parental behaviors have strong associations with a young adult’s mental health, self-worth, and attachment. Further expanding the knowledge of parenting styles and their determinants to child outcomes can only improve our guidance to parents and our service to adolescents and young adults.

## Supporting information

S1 File Contains supplementary methods, supplementary results, supplementary discussion, references, and Tables A-D. (DOCX)
