# Supplementary material for: Relation between parenting style and confident decision-making in a student population
Source: PLoS One. 2024 Nov 12;19(11):e0302495. doi: 10.1371/journal.pone.0302495 (PMC11556737; doi:10.1371/journal.pone.0302495)
Supplement: S1 File — (DOCX) [file pone.0302495.s001.docx]

**S1 Supporting Information**

**Contents**

Supplementary Methods………………………………………...………………………...………2

Participants…………………………………………………………………..………...…..2

Trust Game Explanation………………………………………………..……………..…..2

Exploratory Analysis Plan………………………………………………………..….……3

Supplementary Results……..………………………………………………………..…………….4

Parenting style groups and secondary variables………………..……………………...….4

Correlations between parenting behaviors and secondary variables……………………...5

Parenting behaviors, trust, and reciprocity….…………………………………………….6

Supplementary Discussion………………………………………….……………….…….………7

Parenting, self-esteem, and self-efficacy……………………………………..………...…7

Parenting and mental health……………..……………………………..…….……………9

References……………………………………………………………………………………..…10Table A…………………………….………………………………………………………..……12

Table B…………………………………………….………………………………..……………15

Table C…………………………………………………………….…………………………..…17

Table D…………..……………………………………………………………….....……………18

**Supplementary Methods**

**Participants**

Participants were self-selected and opted to participate online through the department’s Research Experience Program. Students who participated in the experiment received extra credit points for their psychology classes. Students were also provided with the opportunity to participate in a lottery for a gift card ($20) that would be determined after study closure.

The mean age of the sample was 20.69 years (*SD* = 3.14) A majority of the participants were female (78%), white (56%), had been reared by two biological parents (84%), and had identified their mother as their predominant caregiver (88%). Other participants were male (20%) or nonbinary (2%); reared by a single parent (7%), a biological parent and stepparent (5%), adoptive parents (3.5%), or other relative caregiver (0.5%); and had identified their father (9.5%), grandmother (1%), aunt (0.5%), stepfather (0.5%), or adoptive parent (0.5%) as their predominant caregiver (see Table A).

**Trust Game Explanation**

In this version of the game, based off of the original Trust Game [1A] by Berg, Dickhaut, and McCabe, there are two roles: the investor and the trustee. The investor begins with 5 monetary units (MU) and can decide to give a portion of them to the trustee, who starts with zero MU in this rendition. The trustee receives three times the amount sent to them by the investor (if the investor chose to give 5 MU, the trustee receives 15 MU, etc.). The trustee can return some of the MUs received, although they do not have to. For this study, each participant began the online task as the investor to make one investment decision and then switched roles to play as the trustee to make a decision for five hypothetical scenarios. We did not disclose to participants beforehand that they would be playing a trial in each role in the task instructions.

Participants began by completing a pre-task quiz to test their understanding of the game. They decided how much to invest while playing as the investor, then predicted how many MUs would be returned to them had they invested the highest possible amount to the trustee (5 MU, resulting in 15 MU received by the trustee). As the trustee, participants decided how many MUs to return based on five hypothetical scenarios, one for each possible amount they could have received from an investor (3, 6, 9, 12, or 15 MU). Participants then rated their confidence in their choice from 0 (not at all confident) to 10 (completely confident). This occurred six times throughout the task (the first rating occurred while playing as the investor). The ratings completed throughout the Trust Game were averaged to serve as the mean confidence score from this task. Verbatim wording used for the Trust Game, as adapted for this study, can be viewed in our codebook available through the OSF repository (<https://osf.io/xp2ed/?view_only=a21d65fd6292475ba1ee8b2053f76117>) [2A].

Participants were informed that after the study was closed we would randomly assign participants to each other using a random pair generator, pairing an investor role with a trustee role. The response from the trustee was chosen based on the amount the randomly assigned investor chose to send. Participants were awarded MU in the form of lottery tickets (1 MU = 1 lottery ticket).

**Exploratory Analysis Plan**

To explore whether mean ranks of scores from our secondary measures significantly differed between parenting styles (authoritarian/affectionless control, permissive/optimal, authoritative/affectionate constraint) in a manner similar to previous research, we conducted six additional Kruskal-Wallis one-way ANOVAs. These followed the same constraints in our principal analysis, using perceived parenting style as the predictor variable. Scores from our peripheral measures (NGSE, RSES, ACE, DASS-21, and AAQ anxiety and AAQ avoidance scores) served as the dependent variable in each test.

To examine whether PBI care, PBI overprotection, NGSE, RSES, ACE, DASS-21, and AAQ anxiety and AAQ avoidance scores were all correlated with one another, we conducted several additional Spearman correlations.

As a final exploratory test, we operationalized “trust” and “reciprocity” from the Trust Game to examine, by conducting Spearman correlations, whether these scores were significantly related to any others (PBI care, PBI overprotection, NGSE, RSES, ACE, DASS-21, and AAQ anxiety and AAQ avoidance scores). “Trust” scores were represented by the amount of MU the investor gave to the trustee, whereas “reciprocity” scores were operationalized as the number of MU the trustee returned or reciprocated when the investor invested the highest possible amount of MU (15).

**Supplementary Results (Exploratory Analysis)**

**Parenting style groups and secondary variables**

Multiple pairwise comparisons using Dunn’s test (see Table D) found that participants reared by optimal/permissive parenting were significantly different than participants reared by the authoritarian/affectionless control parenting on all secondary variables, with higher mean ranks for self-efficacy (*p* < .001) and self-esteem (*p* < .001) and significantly lower mean ranks for adverse childhood experiences (*p* < .001); depression, anxiety, and stress symptoms (*p* < .001); attachment avoidance (*p* < .001); and attachment anxiety (*p* < .001). All p-values have been adjusted for Bonferroni’s correction for multiple comparisons.

Dunn tests also found that participants reared by the permissive/optimal parenting style had significantly higher mean ranks than the authoritative/affectionate constraint style for self-efficacy (p = .045), and self-esteem (*p* < .001), and significantly lower mean ranks for depression, anxiety, and stress symptoms (*p* < .001); and attachment avoidance (*p* = .029) . Participants reared by these styles did not differ significantly on scores for attachment anxiety (*p* = .081) nor adverse childhood experiences (*p* = .999) .

Lastly, Dunn tests indicated that the mean ranks of scores of participants reared by the authoritative/affectionate constraint parenting style did not differ significantly from participants reared by the authoritarian/affectionless control style on self-esteem (*p* = .158) and depression, anxiety, and stress symptoms (*p* = .312), and attachment anxiety (p = .093) . Participants reared by the authoritative/affectionate constraint style had significantly higher mean ranks on scores for self-efficacy (*p* = .004) and significantly lower mean ranks than those reared by the authoritarian/affectionless control style for adverse childhood experiences (*p* < .001) and attachment avoidance (*p* = .001).

**Correlations between parenting behaviors and secondary variables**

Perceived parental care and overprotection scores were significantly correlated with all variables aside from decision-confidence (see Table 2 in main text). Perceived parental care scores were positively correlated with self-efficacy (*r_s_* = .44, *p* < .001) and self-esteem (*r_s_* = .49, *p* < .001), and negatively correlated with adverse childhood experiences (*r_s_* = -.46, *p* < .001), overall DASS-21 scores (*r_s_* = -.44, *p* < .001), attachment avoidance scores (*r_s_* = -.49, *p* < .001), attachment anxiety scores (*r_s_* = -.33, *p* < .001), and perceived parental overprotection scores (*r_s_* = -.54, *p* < .001). Perceived parental overprotection scores, in contrast, were negatively correlated with self-efficacy (*r_s_* = -.31, *p* < .001) and self-esteem (*r_s_* = -.41, *p* < .001), and positively correlated with adverse childhood experiences (*r_s_* = .32, *p* < .001), overall DASS-21 scores (*r_s_* = .45, *p* < .001), attachment avoidance (*r_s_* = .39, *p* < .001), and attachment anxiety (*r_s_* = .31, *p* < .001).

**Parenting behaviors, trust, and reciprocity**

Trust was operationalized as the amount of MUs the investor entrusted with the trustee. Trust, by this definition, was not significantly correlated with perceived parental care (*r_s_* = -.001, *p* = .99), perceived parental overprotection (*r_s_* = -.09, *p* = .16), self-efficacy (*r_s_* = .04, *p* = .54), self-esteem (*r_s_* = .004, *p* = .95), adverse childhood experiences (*r_s_* = -.05, *p* = .48), overall DASS-21 scores (*r_s_* = -.05, *p* = .42), depressive symptoms (*r_s_* < -.001, *p* = .99), anxiety symptoms (*r_s_* = -.06, *p* = .37), stress symptoms (*r_s_* = -.08, *p* = .19), nor attachment anxiety (*r_s_* = -.06, *p* = .39). A small negative correlation was observed between trust and attachment avoidance (*r_s_* = -.14, *p* = .02), but it wouldn’t survive a Bonferroni correction for multiple comparisons, so it should be also taken as merely suggestive.

Reciprocity was operationalized as the amount of MUs the trustee returned to the investor when the investor invested the highest possible amount of MU (15). Reciprocity was not significantly correlated with any variables: perceived parental care (*r_s_* = -.02, *p* = .75), perceived parental overprotection (*r_s_* = -.12, *p* = .06), self-efficacy (*r_s_* = .005, *p* = .93), self-esteem (*r_s_* = -.07, *p* = .25), adverse childhood experiences (*r_s_* = .10, *p* = .11), overall DASS-21 scores (*r_s_* = .04, *p* = .58), depressive symptoms (*r_s_* = .04, *p* = .55), anxiety symptoms (*r_s_* = -.003, *p* = .96), stress symptoms (*r_s_* = .05, *p* = .43), attachment avoidance (*r_s_* = .009, *p* = .88), nor attachment anxiety (*r_s_* = .10, *p* = .13).

**Supplementary Discussion**

Exploratory analyses confirmed that self-esteem, self-efficacy, adverse childhood experiences, symptoms of psychopathology, and attachment were all associated with perceived parental care and overprotection, as well as confirmed that each of these variables was interrelated with one another. Furthermore, groups differed based on the parenting style of their primary caregiver for each of these developmental outcomes in a manner that reflects previous research.

**Parenting, self-esteem, and self-efficacy**

Although participants’ perception of their caregiver’s parenting behaviors had no relation to their decision-confidence in our study, post hoc comparisons found that participants’ self-esteem and self-efficacy were closely related to how caring or overprotective they viewed their parent. Specifically, participants with high self-esteem and self-efficacy described their parents as warm caregivers, while those who had a poorer perception of themselves labeled their parents as uncaring and overprotective. This supports the suggestion by Wolfradt and colleagues [3A] that social support through a positive relationship with one’s parents might be a protective factor against the development of low self-esteem.

While participants’ level of decision-confidence could not be linked to the categorical parenting-style of their primary caregiver, their attitude towards themselves and their abilities could be. Research has indicated that individuals reared by uncaring and overprotective authoritarian parents have low self-esteem and self-efficacy [4A,5A], and our results confirmed this. We could not resolve whether authoritative (caring and overprotective) parents reared children with greater self-esteem than authoritarian parents, as there was no notable difference between them; yet, both styles appeared to be linked to lower self-esteem compared to permissive parents.

In summary, participants who possessed the greatest amount of self-esteem and self-efficacy identified a permissive/optimal caregiver. Children of authoritative/affectionate constraint parents had lower self-esteem and self-efficacy compared to participants with permissive parents, although had higher levels relative to children of authoritarian/affectionless control parents for self-efficacy only. Our results appear to partly contradict a meta-analysis conducted by Pinquart and Gerke [6A]. While our study also found a negative relation between authoritarian parenting and self-esteem, in Pinquart and Gerke’s meta-analysis, authoritative parents (and not permissive parents) had offspring with the highest self-esteem. This disparity may be due to the fact that most of the studies in their meta-analysis employed the Parental Authority Questionnaire [PAQ; 7A] as the primary measure and not the Parental Bonding Instrument [8A], which we utilized. The PAQ instead uses Baumrind’s framework [9A] to classify parents, based on their child’s responses, using only a dimension of “authority”. Within the scale, authoritative parenting is considered to be the perfect middle, where authoritarian parents exert too much control and permissive too little. However, our study supports the idea that permissive/optimal parenting, as measured by the PBI, may be the most protective against the development of poor self-esteem and other mental health issues in adolescents. Indeed, in their meta-analysis, Pinquart and Gerke found that if a permissive parenting style is defined in terms of both low control (overprotection) but also high warmth (care), then the correlation with self-esteem becomes positive [6A]. Future studies could include both the PBI and the PAQ in analyzing self-esteem related to parenting behaviors to see if results differ based on the questionnaire used.

**Parenting and mental health**

Participants who identified uncaring or overprotective behaviors in their primary caregiver were more likely to disclose mental health issues—including higher rates of depressive, anxious, and stress symptoms; greater attachment avoidance and anxiety; and more negative events experienced in their childhood. Previous research has also linked strict parenting to depression and anxiety, and the inability to create a healthy relationship [3A,10A]. However, one should note that we cannot claim causality in either direction, as this is a cross-sectional correlation study. Furthermore, the majority of our sample consisted of white students from a Western university, so the link between uncaring and overprotective parenting and mental health may not appear as negative in a sample from another culture, race, or age group.

On the other hand, participants with a caring parent were less likely to divulge depressive or anxious symptoms, abuse, or proclivity towards insecure attachment. This is also corroborated by pre-existing studies that show that caring parenting styles are linked with lower rates of psychopathological symptoms [3A,10A]. However, in our study, children of permissive (“optimal”) parents were again associated with the most positive outcomes, with the least amount of depressive, anxious, and stress symptoms. Children of authoritative parents, on the other hand, were not considerably different from the youth of authoritarian parents in their ratings of depressive, anxious, and stress symptoms. It is possible that the overprotection in authoritative parenting (“affectionate constraint”) dampens the influence of caring behavior, and that the warm, rarely overbearing nature of permissive parenting is most beneficial for an adolescent’s mental health as it relates to these symptoms. However, it is worth reiterating that Baumrind’s original idea for an authoritative parenting style did not necessarily include a separate overprotection dimension, and that our finding that the permissive parenting style was the one associated with the least number of psychopathological symptoms may be related to the instrument we used to measure parenting styles: the Parental Bonding Instrument (where the permissive parenting style is called “optimal”).

**References**

1A. Berg J, Dickhaut J, McCabe K. Trust, Reciprocity, and Social History. Games and Economic Behavior. 1995 Jul 1;10(1):122–42.

2A. Wolf K, Tajchman Z, Vilares I. Relation between parenting style and confident decision-making in a student population [Internet]. OSF; 2021 [cited 2023 Sep 14]. Available from:<https://osf.io/xp2ed/?view_only=a21d65fd6292475ba1ee8b2053f76117>

3A. Wolfradt U, Hempel S, Miles JNV. Perceived parenting styles, depersonalisation, anxiety and coping behaviour in adolescents. Personality and Individual Differences. 2003 Feb 1;34(3):521–32.

4A. Herz L, Gullone E. The relationship between self-esteem and parenting style: A cross-cultural comparison of Australian and Vietnamese Australian adolescents. Journal of Cross-Cultural Psychology. 1999;30(6):742–61.

5A. Yousaf S. Parenting Style and Self-efficacy among Adolescents. Research on Humanities and Social Sciences. 2015;5(3).

6A. Pinquart M, Gerke DC. Associations of Parenting Styles with Self-Esteem in Children and Adolescents: A Meta-Analysis. J Child Fam Stud. 2019 Aug 1;28(8):2017–35.

7A. Buri JR. Parental Authority Questionnaire. Journal of Personality Assessment. 1991;57(1):110–9.

8A. Parker G, Tupling H, Brown LB. A Parental Bonding Instrument. British Journal of Medical Psychology. 1979;52(1):1–10.

9A. Baumrind D. Effects of Authoritative Parental Control on Child Behavior. Child Development. 1966;37(4):887–907.

10A. Joshi HL, Sharma M, Mehra RK. Depression among Adolescents: Role of Self Efficacy and Parenting Style. SIS Journal of Projective Psychology & Mental Health. 2009 Jan;16(1):13-7.

**Table A. Participant demographics.**

|  | **Identity** | **Affectionless control/ Authoritarian (*n* = 62)** | **Optimal parenting/ Permissive**  **(*n* = 107)** | **Affectionate constraint/ Authoritative (*n* = 56)** | **Neglectful parenting/ Uninvolved (*n* = 21)** | **Overall**  **Sample**  **(*N* = 246^a^)** |
| --- | --- | --- | --- | --- | --- | --- |
| **Age^b^** |  | 21.03 | 20.04 | 21.27 | 21.43 | 20.69 |
| **Gender** | Male | 13 | 21 | 12 | 3 | 49 |
|  | Female | 47 | 84 | 43 | 17 | 191 |
|  | Nonbinary | 2 | 2 | 0 | 1 | 5 |
|  | Preferred not to say | 0 | 0 | 1 | 0 | 1 |
| **Race** | Native American or Native Alaskan | 0 | 0 | 0 | 0 | 0 |
|  | Asian | 26 | 14 | 11 | 6 | 57 |
|  | African American | 3 | 4 | 5 | 3 | 15 |
|  | Hispanic or Latinx | 0 | 3 | 1 | 1 | 4 |
|  | Middle Eastern or North African | 2 | 1 | 4 | 0 | 7 |
|  | Native Hawaiian or Pacific Islander | 0 | 0 | 0 | 0 | 0 |
|  | White | 24 | 78 | 27 | 9 | 139 |
|  | Two or more identified | 5 | 6 | 7 | 2 | 20 |
|  | Preferred not to say | 2 | 1 | 1 | 0 | 4 |
|  | **Identity** | **Affectionless control/ Authoritarian (*n* = 62)** | **Optimal parenting/ Permissive**  **(*n* = 107)** | **Affectionate constraint/ Authoritative (*n* = 56)** | **Neglectful parenting/ Uninvolved (*n* = 21)** | **Overall**  **Sample**  **(*N* = 246^a^)** |
| **Race** | Preferred not to say | 2 | 1 | 1 | 0 | 4 |
| **Primary caregiver** | Mother | 55 | 95 | 50 | 17 | 217 |
|  | Father | 6 | 10 | 4 | 3 | 23 |
|  | Grandmother | 0 | 0 | 2 | 1 | 3 |
|  | Aunt | 0 | 1 | 0 | 0 | 1 |
|  | Stepfather | 1 | 0 | 0 | 0 | 1 |
|  | Adoptive parent | 0 | 1 | 0 | 0 | 1 |
| **Parent environment** | Two biological parents | 51 | 94 | 46 | 16 | 207 |
|  | Single biological parent | 6 | 5 | 4 | 3 | 18 |
|  | Biological & stepparent | 4 | 3 | 4 | 1 | 12 |
|  | Adoptive parents | 1 | 4 | 2 | 1 | 8 |
|  | Relative caregiver | 0 | 1 | 0 | 0 | 1 |
|  | Non-relative caregiver | 0 | 0 | 0 | 0 | 0 |

*Note.* Parenting style groups determined by the Parental Bonding Instrument (PBI).

^a^The overall sample includes eight individuals who opted out of responding to some or all of the Adverse Childhood Experiences scale, and were excluded from some analyses that used data from the scale. ^b^Age reported in years. The remaining values represent (*n*), the sample size of the group.

**Table B. Descriptive statistics: average score per item on measures with standard deviations**

|  | **Affectionless control/ Authoritarian (*n* = 62)** | **Optimal parenting/ Permissive (*n* = 107)** | **Affectionate constraint/ Authoritative (*n* = 56)** | **Neglectful parenting/ Uninvolved (*n* = 21)** | **Overall (*N* = 246)** |
| --- | --- | --- | --- | --- | --- |
| **Variable** |  |  | **Mean ± *SD*** |  |  |
| Parental care | 1.5 ± 0.5 | 2.7 ± 0.2 | 2.6 ± 0.2 | 1.7 ± 0.4 | 2.3 ± 0.7 |
| Parental overprotection | 1.8 ± 0.5 | 0.6 ± 0.2 | 1.4 ± 0.3 | 0.7 ± 0.3 | 1.1 ± 0.6 |
| Self-efficacy | 3.7 ± 0.7 | 4.2 ± 0.6 | 4.1 ± 0.5 | 3.8 ± 0.6 | 4.0 ± 0.6 |
| Self-esteem | 2.6 ± 0.6 | 3.1 ± 0.5 | 2.8 ± 0.5 | 2.7 ± 0.5 | 2.9 ± 0.6 |
| ACE^a^ | 3.4 ± 2.5 | 1.0 ± 1.7 | 1.9 ± 3.0 | 2.2 ± 2.5 | 1.8 ± 2.5 |
| DASS-21 symptoms | 1.2 ± 0.7 | 0.6 ± 0.5 | 1.0 ± 0.7 | 0.9 ± 0.6 | 0.8 ± 0.7 |
| DASS-21 depression | 1.2 ± 0.9 | 0.5 ± 0.6 | 0.9 ± 0.8 | 0.9 ± 0.6 | 0.8 ± 0.8 |
| DASS-21  Anxiety | 1.0 ± 0.7 | 0.4 ± 0.5 | 0.9 ± 0.8 | 0.6 ± 0.6 | 0.7 ± 0.7 |
| DASS-21  Stress | 1.4 ± 0.7 | 0.7 ± 0.6 | 1.1 ± 0.7 | 1.1 ± 0.7 | 1.0 ± 0.7 |
| AAQ avoidance | 4.2 ± 1.1 | 3.1 ± 1.0 | 3.6 ± 1.2 | 4.1 ± 1.0 | 3.6 ± 1.2 |
| AAQ anxiety | 4.1 ± 1.1 | 3.3 ± 1.0 | 3.7 ± 1.0 | 3.8 ± 1.2 | 3.6 ± 1.1 |
| Decision-  confidence | 5.9 ± 2.0 | 5.9 ± 2.4 | 5.7 ± 2.6 | 6.1 ± 2.5 | 5.9 ± 2.3 |

*Note.* Parenting style groups determined by the Parental Bonding Instrument (PBI). Average per item scores taken from the following measures: the Parental Bonding Instrument (perceived care and overprotection subscales, 0-3 Likert scale); the New General Self-Efficacy scale (5-pt Likert scale); Rosenberg Self-Esteem Scale (4-pt Likert scale); the Adverse Childhood Experiences scale (reported value represents the number of ACEs); the Depression, Anxiety, and Stress Scale-21 (“DASS-21 symptoms” reports average per item scores on the entire measure, while individual depression, anxiety, and stress symptom average per item scores are also listed, all use 0-3 Likert scale); the Adult Attachment Questionnaire (attachment avoidance and anxiety subscales, 7-pt Likert scale); and decision-confidence as measured in this study (rated 0-10).

^a^The Adverse Childhood Experiences scale reports the average sum score for each group, contrary to the rest of the measures, as it is the only measure not scored on a Likert scale (1 pt for “yes”, 0 for “no”, 17 pts possible). ^b^The ACE average scores omit data from eight participants who skipped questions from the measure, such that N = 238 for the overall sample (see Appendix F).

**Table C. Visualization of participants who opted out of the Adverse Childhood Experiences scale arranged by perceived parenting style group.**

|  | **Affectionless control/ Authoritarian (*n* = 62)** | **Optimal parenting/ Permissive (*n* = 107)** | **Affectionate constraint/ Authoritative (*n* = 56)** | **Neglectful parenting/ Uninvolved (*n* = 21)** | **Overall**  **Sample**  **(*N* = 246)** |
| --- | --- | --- | --- | --- | --- |
| Nonrespondents | 4 | 1 | 2 | 1 | 8 |
| Respondents | 58 | 106 | 54 | 20 | 238 |

*Note.* Parenting style groups determined by the Parental Bonding Instrument (PBI). Most nonrespondents skipped three items or less. Two nonrespondents skipped the entire questionnaire (one with an affectionless control/authoritarian caregiver, and one with a neglectful/uninvolved caregiver). Nonrespondents were excluded via pairwise deletion from tests that used scores from the Adverse Childhood Experiences scale.

In the exploratory Kruskal-Wallis test that examined whether mean ranks of scores from the Adverse Childhood Experiences scale differed between perceived parenting styles, seven non-respondents were removed via pairwise deletion such that *N* = 218 for this test (the eighth non-respondent was the individual reared by a neglectful parent, and was already not represented in this test; see the table above). The remaining tests utilizing ACE scores removed the eight individuals via pairwise deletion such that *N* = 238 for those tests.

**Table D. Post hoc multiple comparisons Dunn’s test table depicting significant differences between parenting style groups on measures.**

| **Measure** | **Parenting style**  **(a)** | **Parenting style**  **(b)** | ***Z*** | **Adjusted *p-*value**^a^ |
| --- | --- | --- | --- | --- |
| **NGSE** | Optimal | Affectionate Constraint | 2.44 | .045 |
|  |  | Affectionless Control | 5.36 | <.001 |
|  | Affectionless Control | Affectionate Constraint | -2.47 | .048 |
| **RSES** | Optimal | Affectionate Constraint | 3.64 | <.001 |
|  |  | Affectionless Control | 6.00 | <.001 |
|  | Affectionless Control | Affectionate Constraint | -1.94 | .158 |
| **ACE^b^** | Optimal | Affectionate Constraint | -0.88 | .999 |
|  |  | Affectionless Control | -6.11 | <.001 |
|  | Affectionless Control | Affectionate Constraint | 4.50 | <.001 |
| **DASS-21** | Optimal | Affectionate Constraint | -3.91 | <.001 |
| **overall** |  | Affectionless Control | -5.92 | <.001 |
|  | Affectionless Control | Affectionate Constraint | 1.63 | .312 |
| **DASS-21** | Optimal | Affectionate Constraint | -3.14 | <.001 |
| **depression** |  | Affectionless Control | -5.23 | <.001 |
|  | Affectionless Control | Affectionate Constraint | 1.72 | .259 |
| **DASS-21** | Optimal | Affectionate Constraint | -3.90 | <.001 |
| **anxiety** |  | Affectionless Control | -5.48 | <.001 |
|  | Affectionless Control | Affectionate Constraint | 1.26 | .624 |
| **DASS-21** | Optimal | Affectionate Constraint. | -3.80 | <.001 |
| **stress** |  | Affectionless Control | -5.76 | <.001 |
|  | Affectionless Control | Affectionate Constraint. | 1.59 | .337 |
| **Measure** | **Parenting style**  **(a)** | **Parenting style**  **(b)** | ***Z*** | **Adjusted *p-*value** |
| **AAQ** | Optimal | Affectionate Constraint | -2.59 | .029 |
| **avoidance^c^** |  | Affectionless Control | -6.05 | <.001 |
|  | Affectionless Control | Affectionate Cons. | 2.92 | .010 |
| **AAQ** | Optimal | Affectionate Constraint | -2.21 | .080 |
| **anxiety^c^** |  | Affectionless Control | -4.78 | <.001 |
|  | Affectionless Control | Affectionate Constraint | 2.16 | .093 |

*Note*. Parenting styles are from the Parental Bonding Instrument and refer to individuals reared by affectionate constraint (authoritative), optimal (permissive), and affectionless control (authoritarian) parenting styles. Those reared by the neglectful parenting style (uninvolved) are not represented in this table as our study did not obtain a large enough sample to include it in this analysis. Measures: New General Self-Efficacy scale; Rosenberg Self-Esteem Scale; Adverse Childhood Experiences scale; Depression, Anxiety, and Stress Scale-21 (overall score as well as depression, anxiety, and stress symptoms subscale scores); and Adult Attachment Questionnaire (attachment avoidance and anxiety subscales).


^a^Adjustments were made using Bonferroni’s correction for multiple comparisons. ^b^Participants were able to skip the Adverse Childhood Experiences scale due to the sensitive nature of its questions. The data from these participants (seven; the eighth person to opt out was reared by a neglectful/uninvolved parent and thus was already excluded for this test) were removed via pairwise deletion for this exploratory one-way Kruskal-Wallis ANOVA such that *N* = 218. **^c^**Scores obtained from the AAQ subscales represented normal distributions and equal variances, but nonparametric Kruskal-Wallis tests were performed with this data as well for consistency.
